# Sustained impact of an antibiotic stewardship initiative targeting asymptomatic bacteriuria and pyuria in the emergency department

**DOI:** 10.1017/ash.2022.289

**Published:** 2022-08-30

**Authors:** Mary Catherine Cash, Garrett Hile, James Johnson, Tyler Stone, Jessica Smith, Chris Ohl, Vera Luther, James Beardsley

**Affiliations:** 1 Department of Pharmacy, Wake Forest Baptist Health, Winston-Salem, North Carolina; 2 Department of Pharmacy, University of Kentucky HealthCare, Lexington, Kentucky; 3 Section on Infectious Diseases, Department of Internal Medicine, Wake Forest School of Medicine, Winston-Salem, North Carolina; 4 Department of Pharmacy, The Ohio State University Wexner Medical Center, Columbus, Ohio

## Abstract

**Objective:**

To determine whether a multifaceted initiative resulted in maintained reduction in inappropriate treatment of asymptomatic pyuria (ASP) or bacteriuria (ASB) in the emergency department (ED).

**Design:**

Single-center, retrospective study.

**Methods:**

Beginning in December 2015, a series of interventions were implemented to decrease the inappropriate treatment of ASP or ASB in the ED. Patients discharged from the ED from August to October 2015 (preintervention period), from December 2016 to February 2017 (postintervention period 1), and from November 2019 to January 2020 (postintervention period 2) were included if they had pyuria and/or bacteriuria without urinary symptoms. The primary outcome was the proportion of patients prescribed antibiotics within 72 hours of discharge from the ED. The secondary outcome was the number of patients returning to the ED with symptomatic UTI within 30 days of discharge.

**Results:**

We detected a significant decrease in the proportion of patients with ASP or ASB who were inappropriately treated when comparing the preintervention group and post-intervention group 1 (100% vs 32.4%; *P* < .001). This reduced frequency of inappropriate treatment was noted 3 years after the intervention, with 28% of patients receiving treatment for ASP or ASB in postintervention group 2. (*P* was not significant fin the comparison with postintervention group 1.) Among the 3 groups analyzed, we detected no difference in the numbers of patients returning to the ED with a symptomatic UTI within 30 days of ED discharge regardless of whether patients received antibiotics.

**Conclusions:**

A multifaceted intervention resulted in a significant decrease in inappropriate use of antibiotics for ASP and/or ASB that was maintained 3 years after implementation.

Urinary tract infection (UTI) is one of the most common bacterial infections in the United States, resulting in an estimated 2 million visits to emergency departments (EDs) each year.^1^ Symptoms of UTI include urinary urgency and frequency, suprapubic tenderness, costovertebral angle tenderness, and fever. Transient bacteriuria or colonization may result in asymptomatic bacteriuria (ASB), defined as the isolation of bacteria from an appropriately collected urine sample in a patient who lacks signs and symptoms of a UTI. Because ASB is not reflective of true infection, current Infectious Diseases Society of America (IDSA) guidelines recommend against the use of antibiotics to treat ASB except in the case of pregnancy or planned urologic procedure.^2^ Furthermore, it has been suggested that the use of antibiotics to treat ASB results in potentially avoidable adverse drug events including increased antibiotic resistance, increased rates of *Clostridioides difficile* infection, and increased rates of recurrent symptomatic UTI.^3–5^ Despite guideline recommendations against the use of antibiotics and the demonstrated risk of inappropriate antibiotic use, treatment of ASB remains common in clinical practice.^6,7^

In recent years, initiatives have been made to reduce the overprescribing of antibiotics in asymptomatic patients.^9–19^ Many studies have demonstrated an improvement in prescribing practices following educational intervention. Strategies for these interventions ranged from a single 1-hour educational session to the development of algorithms and pocket cards to assist prescribers and nursing staff in decision making. Not only have pharmacist-led educational interventions been shown to decrease the rate of inappropriate treatment of ASB, but some studies have also suggested a cost benefit associated with minimization of unnecessary treatment.^14,20^

Asymptomatic pyuria (ASP), defined as a urine specimen that contains increased numbers of leukocytes in an otherwise asymptomatic patient, represents the presence of inflammation that is not always due to true infection. However, it also commonly results in the inappropriate initiation of antibiotics.^8^ Although ASB has gained recognition in recent years for promoting inappropriate antibiotic use, the impact of ASP on prescribing behavior and the impact antimicrobial stewardship initiatives have on those behaviors is not well characterized.

Several studies have demonstrated improvement in prescribing practices immediately following educational interventions, but few have characterized the sustained impact of such antimicrobial stewardship initiatives. Similarly, few studies have formally evaluated the influence of ASP on prescribing in the ED or the effects of initiatives to improve such prescribing. Therefore, the purpose of this study was to determine the sustained impact of a multifaceted intervention on the rate of inappropriate treatment of ASP or ASB in the ED at our institution.

## Methods

This single-center, retrospective study was conducted at the Wake Forest Baptist Medical Center (WFBMC) ED. The 45-bed adult ED at WFBMC receives ∼80,000 visits annually. The study was approved by the Wake Forest Baptist Health institutional review board.

**Fig. 1.**
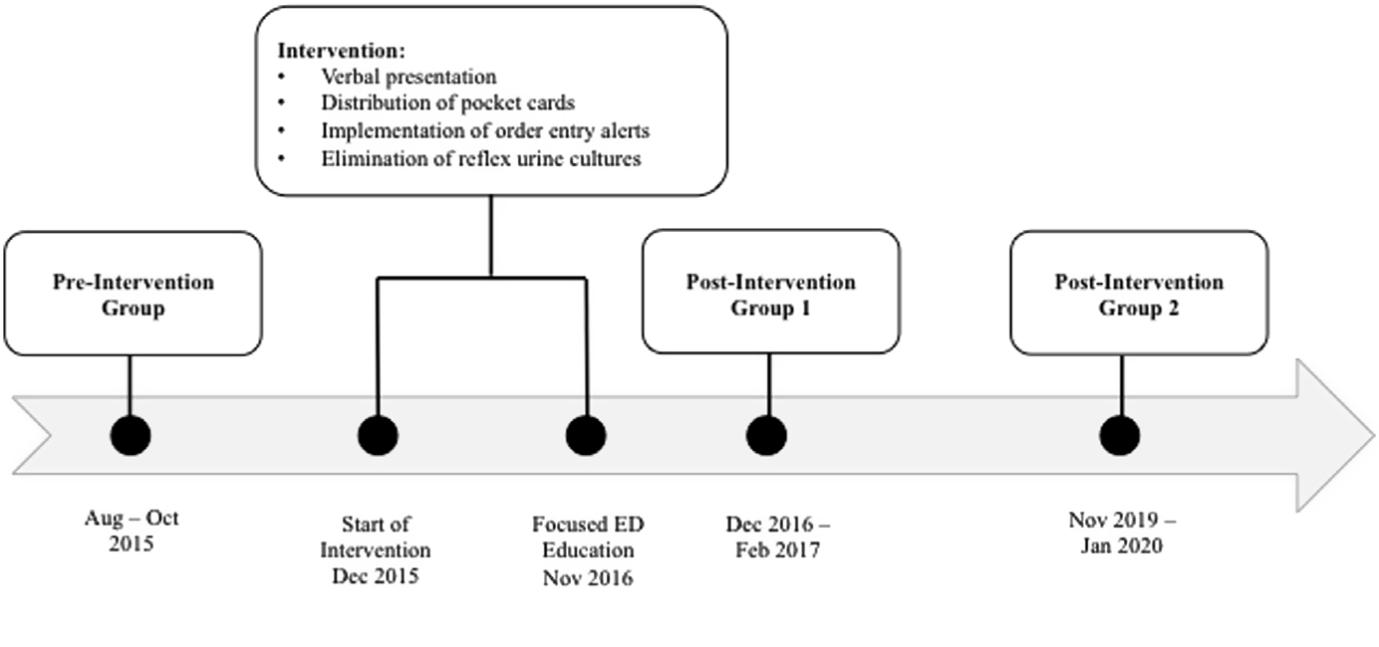
Timeline of intervention in relation to study groups.

Beginning in December 2015, a series of interventions were implemented to decrease the unnecessary treatment of ASB and ASP at the study institution. Interventions included verbal presentations to physicians and pharmacists, distribution of pocket cards and treatment algorithms (Supplementary Fig. 1), alerts embedded into order-entry software addressing the appropriate use of urine cultures (Supplementary Fig. 2), and elimination of reflex urine-culture orders for positive urinalyses (Fig. 1). These interventions took place over several months and targeted providers in the ED as well as internal medicine service lines. In November 2016, ED pharmacists assisted with a focused education that targeted practices in the ED. These interventions were implemented in the setting of a well-established antimicrobial stewardship program and 24-hour ED pharmacist coverage. To examine both the immediate and long-term effects of these interventions, the investigators chose a preintervention group that included patients discharged from the ED prior to the implementation of any interventions, a postintervention group 1 that included patients discharged from the ED in the months immediately following the focused education in the ED, and a postintervention group 2 that included patients discharged from the ED 3 years after the focused education.

Patients who had urine studies completed prior to discharge from the ED were identified using the electronic medical record (EMR) database (Epic Systems, Verona, WI). Results were filtered to identify patients who had urine study results meeting defined study criteria. Identified patients were then retrospectively screened for inclusion. Patients were reviewed consecutively in the chronological order in which they received care until the target sample size in each group was attained.

Patients were eligible for inclusion if they were at least 18 years of age and were discharged from the ED between August and October 2015 (preintervention group), between December 2016 and February 2017 (postintervention group 1), or between November 2019 and January 2020 (postintervention group 2) (Fig. 1). Patients were included if urine studies collected during the ED visit resulted in either a urinalysis with >12 white blood cells per high-power field or a urine culture with ≥100,000 colony-forming units per milliliter. Patients were excluded (1) if they had signs or symptoms of UTI including urinary frequency or urgency, dysuria, suprapubic tenderness, or temperature >100.3°F or 37.9°C according to EMR documentation; (2) if they were pregnant; (3) if they had an indwelling catheter, ureteral stent, or nephrostomy tube; (4) if the had been were diagnosed with another infection requiring treatment with antibiotics; and/or (5) if they were immunocompromised. Immunocompromised status was defined as human immunodeficiency virus with CD4 count < 50 cells/mm^3^, history of stem-cell or solid-organ transplant, use of prednisone 20 mg equivalent of a glucocorticoid for at least 2 weeks, therapy with any medication used for prevention of organ rejection, or an absolute neutrophil count <1,000 cells/mm^3^.

The primary outcome was the proportion of patients with ASP or ASB prescribed antibiotics within 72 hours of ED discharge. The secondary outcome was the number of patients returning to the ED with symptomatic UTI within 30 days of discharge. The preintervention group was compared to postintervention group 1 to determine the intervention’s immediate impact. Postintervention group 1 was compared to postintervention group 2 to determine the sustainability of the initiative.

We used the χ^2^ or Fisher exact test to analyze categoric data. A sample size calculation predicted that 32 patients in the preintervention group and in postintervention group 1 would provide 80% power at an α of 0.05 to detect a 30% relative reduction in the inappropriate treatment of ASB and ASP. Target sample size was set at 37 patients for each of these groups. The inclusion of 50 patients in postintervention group 2 would provide 80% power at an α of 0.05 to detect an absolute increase of 25% in the proportion of inappropriate prescribing for ASB and/or ASP when compared to postintervention group 1.

## Results

**Fig. 2.**
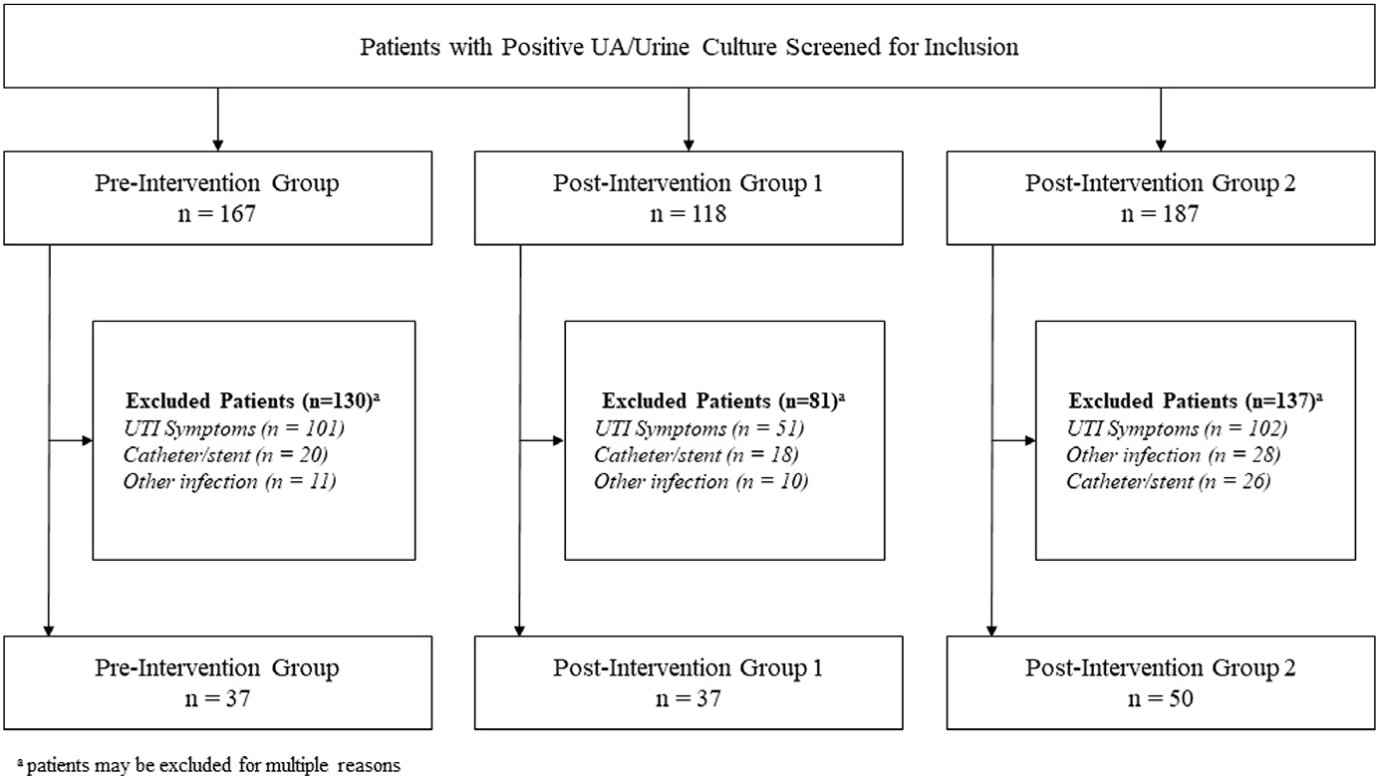
Inclusion and exclusion.

In the preintervention group, 167 patients were screened to identify 37 with ASB or ASP for whom antibiotic treatment was not indicated. In the postintervention group 1, 118 patients were screened to identify 37 patients for inclusion. In the postintervention group 2, 187 patients were screened until 50 were included. The most common reasons for exclusion were documented signs or symptoms of UTI, presence of another infection requiring antibiotics, and presence of an indwelling catheter, ureteral stent, or nephrostomy tube (Fig. 2). Baseline characteristics were similar among groups (Table 1).

**Table 1.** Patient Characteristics

Variable	Preintervention Group (N = 37)	Postintervention Group 1 (N = 37)	Postintervention Group 2 (N = 50)
Age, mean y (SD)	50.6 (22.5)	41.8 (23)	49.9 (24.6)
Sex, female, no. (%)	27 (73)	34 (91.9)	43 (86)

**Fig. 3.**
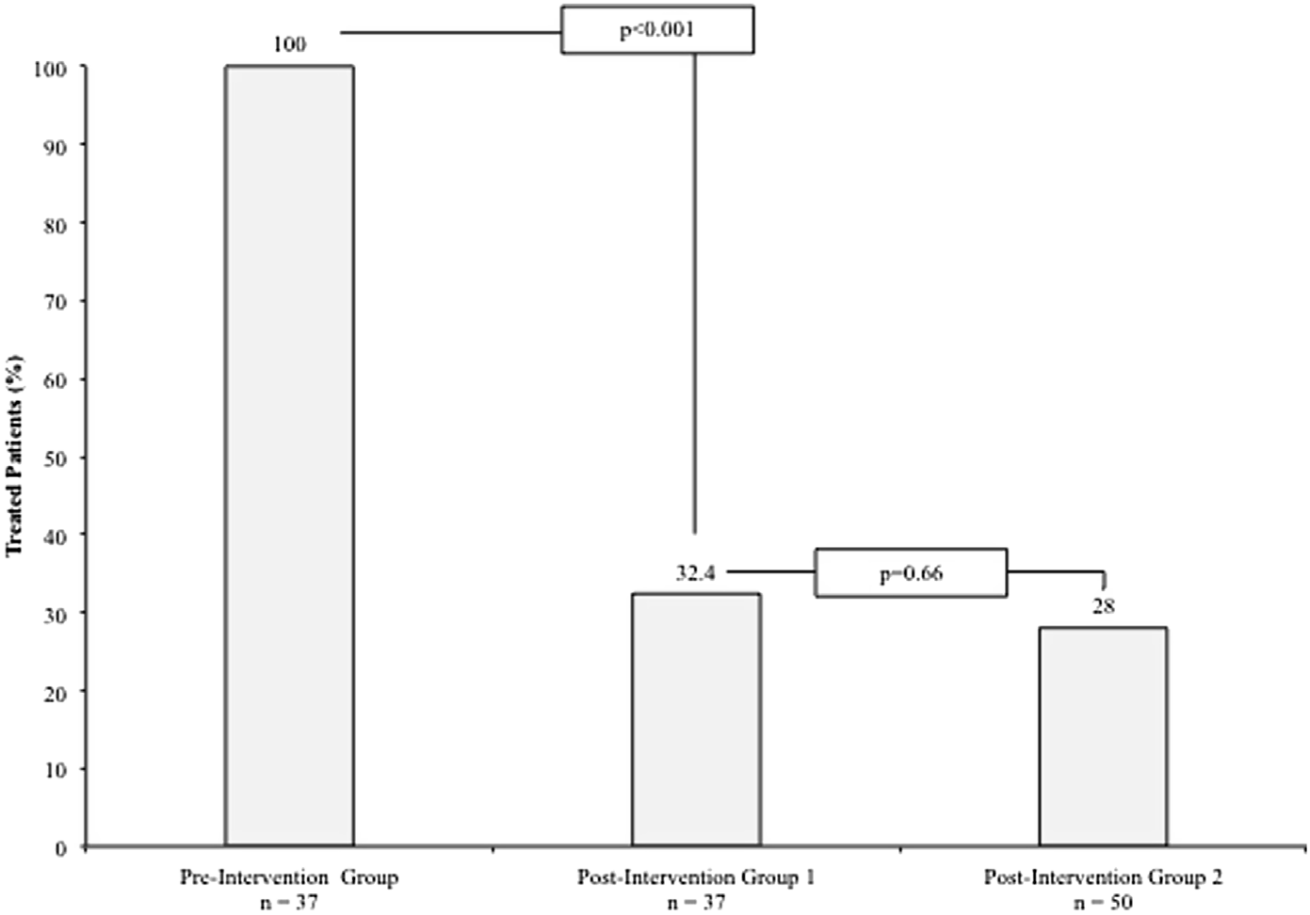
Proportion of patients inappropriately treated for ASP and/or ASB.

The intervention resulted in a dramatic reduction in inappropriate prescribing for ASP or ASB from 100% in the preintervention group to 32.4% in postintervention group 1 (*P* < .001) (Fig. 3). No change in the proportion of inappropriate prescribing was observed 3 years after the intervention, with 28% of patients receiving treatment for ASP and/or ASB in post-intervention group 2. *P* was not significant in the comparison with postintervention group 1 (Fig. 3).

Among those treated for ASB or ASP in the preintervention group, 25 (68%) were treated based on ASP in the absence of bacteriuria. This inappropriate antibiotic use in response to ASP was reduced in the months immediately following implementation of the intervention: 100% in the preintervention group versus 22% in postintervention group 1 (*P* < .001) (Table 2). No change in the proportion of inappropriate prescribing for ASP was observed years after the intervention, and 10 patients (21.7%) in postintervention group 2 received treatment. *P* was not significant in the comparison with postintervention group 1 (Table 2).

**Table 2.** Immediate and Sustained Impact of a Multi-faceted Stewardship Initiative

Patients Inappropriately Prescribed Antibiotics	Preintervention Group (N = 37), No. (%)	Postintervention Group 1 (N = 37), No. (%)	Postintervention Group 2 (N = 50), No. (%)
All patients	37/37 (100)	12/37 (32.4)^a^	14/50 (28)
Patients with ASP alone	25/25 (100)	6/27 (22.2)^a^	10/46 (21.7)
Patients with ASB alone	5/5 (100)	3/4 (75)	0/0 (0)
Patient with ASP + ASB	7/7 (100)	3/6 (50)	4/4 (100)
Patients returning to ED for UTI within 30 d	3^b^	4^c^	1^d^

Note. ASP, asymptomatic pyuria; ASB, asymptomatic bacteriuria.

a *P* < .05 for comparison with preintervention group.

b 3 of 3 had received antibiotics for ASB and/or ASP.

c 2 of 4 had received antibiotics for ASP and/or ASB.

d 1 of 1 had received antibiotics for ASP and/or ASB.

Furthermore, 3 patients in the preintervention group, 4 patients in postintervention group 1, and 1 patient in postintervention group 2 returned to the ED with a symptomatic UTI within 30 days (Table 2). Withholding antibiotics for ASB or ASP did not result in an increased risk of returning with a symptomatic UTI; 2 (3%) of 61 untreated patients and 6 (10%) of 63 treated patients returned with a symptomatic UTI.

## Discussion

The multifaceted antimicrobial stewardship initiative resulted in a significant decrease in the inappropriate treatment of ASP or ASB in the ED in the 3 months immediately following its implementation. Furthermore, this reduction was still noted when evaluated 3 years after the initial intervention.

The treatment rate in the preintervention group (100%) was higher than predicted, emphasizing the importance of these interventions. Treatment rates decreased by approximately two-thirds in postintervention group 1, demonstrating an impact from the initiative that was both statistically and clinically significant. No other changes apart from the interventions could be identified as affecting this outcome.

The sustainability of various antibiotic stewardship initiatives has not been well described in the literature. A controlled crossover study evaluating the timing and duration of antimicrobial surgical prophylaxis demonstrated an improvement in appropriate prescribing practices immediately after education. However, such improvements were not sustained over the course of 12 months.^21^ In contrast, an educational intervention in one nursing home demonstrated a reduction in the submission of inappropriate urine cultures and a reduction in ASB treatment up to 30 months after the intervention.^11^

The maintained reduction in inappropriate treatment seen in this study is likely to have been influenced by several factors. First, alerts embedded into order-entry software, and the elimination of reflex urine culture orders implemented as part of the initiative have remained in place since their implementation. Thus, any impact these interventions had immediately following their implementation was likely to be maintained. The antimicrobial stewardship program at the study institution is strong, which helped optimize the utilization of antimicrobial agents across the medical center, including in the ED. Furthermore, 24-hour pharmacist coverage in the ED may have contributed to day-to-day stewardship interventions that minimized inappropriate treatment throughout the study period.

To our knowledge, this is the first study to demonstrate the impact of ASP on the inappropriate prescribing of antibiotics in the ED. Most patients meeting study inclusion criteria in all groups had ASP in the absence of confirmed ASB, suggesting that ASP may be a driver of inappropriate antibiotic usage. Although the high rate of ASP alone in the preintervention group was surprising, the rate of ASP in the absence of bacteriuria in the postintervention groups was not unexpected as the elimination of reflex urine cultures for positive urinalyses is suspected to have decreased the number of urine cultures overall. Notably, the sustained decrease in the primary outcome appears to have been driven by a sustained decrease in the treatment of ASP. Despite this, most patients treated for ASP did not have urine cultures obtained, suggesting that prescribers are making a presumptive diagnosis of UTI based only on a nonspecific inflammatory marker.

The number of patients returning to the ED with signs or symptoms of UTI within 30 days of discharge was not significantly different among the 3 study groups. However, most of those who did return to the ED with a symptomatic UTI had received antibiotics for ASP and/or ASB. These results suggest that there is no apparent harm from refraining from treatment of asymptomatic patients.

This study had several limitations. First, it was conducted at a single center and included a relatively small sample size. Due to the retrospective nature of the study, data capture was dependent upon accurate documentation in the medical record. Furthermore, exclusion criteria did not account for those with frequent UTIs or other comorbid conditions that may influence prescribing patterns. Finally, the data presented only represent snapshots of antibiotic utilization at various points over 4.5 years. As such, conclusions cannot be made as to the proportion of inappropriate treatment in the interim periods, and advances in medical trainee knowledge throughout the year may have influenced these results.

In conclusion, a multifaceted stewardship initiative significantly decreased the proportion of inappropriate treatment of ASP or ASB in the ED in the months immediately following the interventions. The decreased proportion of inappropriate treatment of ASP and ASB was preserved when it was evaluated 3 years after the initial intervention.

## Supplementary material

For supplementary material accompanying this paper visit https://doi.org/10.1017/ash.2022.289. click here to view supplementary material
