# Supplementary figures and images for: Sustained impact of an antibiotic stewardship initiative targeting asymptomatic bacteriuria and pyuria in the emergency department
Source: Antimicrob Steward Healthc Epidemiol. 2022 Aug 30;2(1):e148. doi: 10.1017/ash.2022.289 (PMC9726488; doi:10.1017/ash.2022.289)

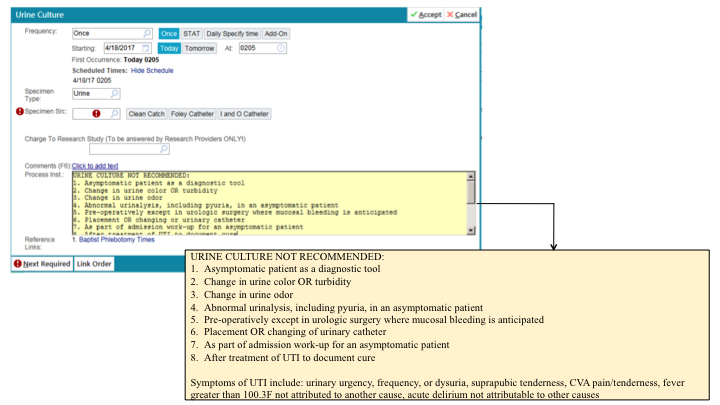

Supplement: Supplementary file 1 [file ashsup.zip › S2732494X22002893sup001.tiff]

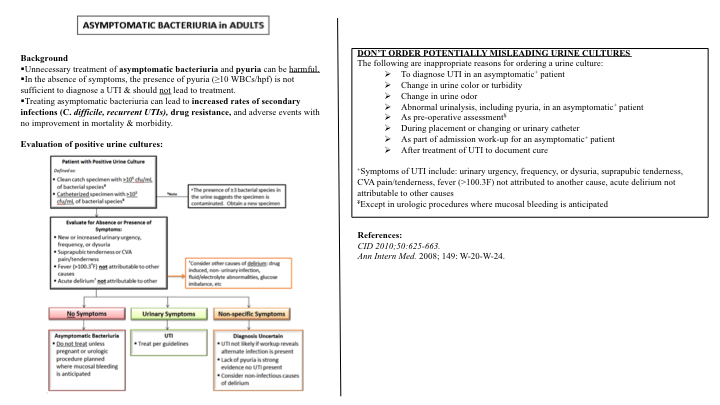

Supplement: Supplementary file 1 [file ashsup.zip › S2732494X22002893sup002.tiff]
